# “Why have I not been told about this?”: a survey of experiences of and attitudes to advance decision-making amongst people with bipolar

**DOI:** 10.12688/wellcomeopenres.14989.2

**Published:** 2019-04-23

**Authors:** Guy Hindley, Lucy A. Stephenson, Alex Ruck Keene, Larry Rifkin, Tania Gergel, Gareth Owen

**Affiliations:** 1 Institute of Psychiatry, Psychology and Neuroscience, King’s College London, London, London, SE5 8AB, UK; 2 39 Essex Chambers, London, WC2A 1DD, UK; 3 South London and Maudsely NHS Foundation Trust, London, SE5 8AZ, UK

**Keywords:** Bipolar, survey, advance decision making, mental capacity, human rights

## Abstract

**Background: **The idea that people with severe mental illness should be able to plan in advance for periods of illness as a means of enhancing autonomy has been long debated and is increasingly being enshrined in codes of practice and mental health legislation. It has been argued that the ethical imperative for this is especially pronounced in bipolar (BP), a condition in which sufferers often experience episodic crises interspersed with periods of wellness. However, there is a paucity of published research investigating experiences of advance decision making (ADM) in people with BP or their attitudes towards it.

**Methods: **An online survey of BPUK’s mailing list was conducted. 932 people with BP completed the survey (response rate 5.61%). Descriptive statistics and regression analysis were conducted to compare experience of with attitudes towards ADM and variables associated with interest in ADM.

**Results: **A majority indicated a desire to plan care in advance of losing capacity (88%) but most had not done so (64%). High numbers of respondents expressed a wish to request as well as refuse treatment and most wanted to collaborate with psychiatrists, including on issues around self-binding. The most frequent motivation to utilise ADM was a desire to be more involved in mental health decisions. Interest in self-binding was associated with experience of compulsory treatment and trust in mental health services. Interest in refusals of all medication was associated with younger age and lack of trust in mental health services. Interest in ADM in general was associated with younger age but not educational level, ethnicity or gender.

**Conclusions: **This study demonstrates an appetite for ADM amongst people with bipolar that is independent of educational status and ethnicity. As states reform their mental health laws, attention needs to be given to the distinctive attitudes toward ADM amongst people with bipolar.

## Abbreviations

BP – Bipolar

ADM – Advance decision making

AD – Advance decision

MCA – Mental Capacity Act 2005

ADRT – Advance decision to refuse treatment

LPA – Lasting power of attorney

MHA – Mental Health Act 1983

SBD – Self-binding directive

BPUK – Bipolar UK

## Introduction

Bipolar (BP) is a common and severe mental illness ^1^. It has a worldwide prevalence of approximately 1% and is associated with 10–20 years shorter life expectancy ^2, 3^. Given its preponderance to present in adolescence and early adulthood and persist throughout a person’s life course, it carries a substantial future burden of disease ^4, 5^.

A defining feature of BP is its fluctuating course, characterised by marked and prolonged changes in mood and energy levels interspersed between periods of wellness ^1^. These episodic “crises”, which are described as either manic, depressive or mixed depending on the predominant polarity of affect, occur relatively frequently. Angst *et al.* reported a median of 1 episode every 2.5 and 3 years for BP I and II patients respectively ^6^. Episodes can bring an array of destructive sequelae. During a manic phase this may include substance misuse, overspending, self-harm and psychotic features ^6^. In order to prevent harm from such episodes, inpatient psychiatric admission and medication may be indicated. However, episodes often lead to the loss of capacity to make such treatment decisions (hereby referred to as capacity) and individuals may uncharacteristically refuse treatment and disengage with services when unwell ^7^.

### Decision-making capacity and advance decision-making

Capacity for treatment is a central (although not universal) concept in current, international, medico-legal thinking which has applications across medical, psychiatric and social care settings. Based upon the principle of self-determination, it demands that a person’s decision is respected so long as it is made with capacity. If the individual has been judged to lack capacity, various mechanisms have been proposed to either make a decision that most closely represents the individual’s wishes or to act in the person’s best interests ^8, 9^.

For scenarios where an individual can anticipate that capacity will be lost and they have particular views on treatment or other issues, advance decision-making (ADM) can be employed. ADM is a broad term that encompasses both legally binding decisions as well as non-legally binding plans and may include information about specific decisions, more generic information that can inform best interest decisions or the designation of a substitute decision maker. ADM may be communicated verbally or in writing, resulting in a document that is sometimes referred to as an advance directive or advance care plan. ADM may be undertaken by the individual alone or in collaboration with family, friends and/or professionals ^10^. It can also contain information about various aspects of an individual’s life such as medical treatment, financial affairs or arrangements for work. This study focuses primarily on medical treatment.

### Legal provision for advance decision making in England and Wales

In England and Wales, the Mental Capacity Act 2005 (MCA) details criteria for determining whether a person has capacity to decide upon a matter, and the process to follow when this is judged to be lacking. This is applicable to any situation in which consent is required from an individual. Lack of capacity refers to decision making inabilities due to any condition which may affect the working of the mind or brain, meaning it is not specific to severe mental illness. In situations where capacity is lost, the MCA has provisions for ADM in the form of an advance decision to refuse treatment (ADRT), an advance statement of wishes and preferences or the appointment of a lasting power of attorney (LPA) ^11^.

There is a second piece of legislation in England and Wales, the Mental Health Act 1983 (MHA), that enables compulsory treatment for mental disorder. This is of particular relevance to people with mental illness such as BP, as they are far more likely to be treated under the MHA than the MCA (although if they were to be treated for a physical health condition, the MCA would still apply). The MHA does not use a capacity-based system to determine the need for compulsory treatment, rather focusing on the degree of risk of harm to the individual or others. Furthermore, in terms of inpatient treatment, it does not currently have any statutory provisions for ADM beyond the refusal of non-urgent electroconvulsive therapy.

The disparity between provisions for ADM under the MCA and the MHA and, by association, people with and without mental illness, is two-fold. Firstly, as described above, the MCA has various mechanisms for ADM inbuilt while the options are far more limited under the MHA. Secondly, if an individual with a mental illness attempts to make a legally-binding advance plan using the MCA (e.g. ADRT), the MHA can be used to overrule it. In the MHA Code of Practice health professionals are advised to take the wishes of the person being detained into account. However, there is no provision for this within the MHA itself. To some people with mental illness and professionals this is disempowering and discriminatory.

### Autonomy (Self-determination) and ADM

People living with BP (and other mental illnesses) may experience the loss of a sense of autonomy, or the ability to act autonomously at multiple levels. Firstly, they may, in retrospect experience periods of illness as inauthentic i.e. not arising from their true sense of self. Secondly, during periods of illness an individual may experience a loss of self-control, engaging in behaviours they would never consider when well. Thirdly, treatment may involve coercive measures. There is pressure from the international human rights community to ensure that those with disabilities have access to social and legal resources which support autonomy, equal to those without disabilities. The United Nations Convention of the Rights of Persons with Disabilities asserts that those with disabilities (including mental disabilities) should be able to exercise control, equal to that of people without disabilities, over all areas of their lives including the management of the disability itself ^12^. ADM is seen as one tool by which this aspiration could be achieved. Within a European context, the Council of Europe (2009) recommends that ‘States should promote self-determination for capable adults in the event of their future incapacity, by means of continuing powers of attorney and advance directives to promote self-determination where future incapacity is anticipated’ ^13^. It is of note that the United Nations Committee on the Rights of Persons with Disabilities does not approve a model which relies on an assessment of capacity to determine whether an individual’s choices are respected. Instead they advocate for the use of supported decision making and understand ADM to be a means to this end ^14^.

The potential for ADM to support autonomous decision making relies on the notion of precedent autonomy ^15^. This is the concept that a person’s prior preferences expressed when capacitous be given precedence over preferences expressed at a later time, when lacking in capacity ^16^. In a mental health setting, particular ethical controversies arise as people may wish their advance preferences around external coercion to be respected during a crisis. For example, they may request early intervention and hospital admission in advance yet during a crisis refuse it. A full discussion of the ethical issues is beyond the scope of this paper. Suffice to say that some, including the authors of this paper, maintain that enabling people with BP to use ADM which requests coercion is an autonomy-maximising measure ^16^.

### Advance decision-making and bipolar

Within the medical profession, ADM has been widely discussed in relation to end-of-life care and life-limiting conditions such as dementia. For example, a PubMed search of ADM and dementia generates 426 citations, compared to BP’s 14, and includes several systematic reviews ^17– 19^. However, one of the philosophical criticisms of ADM for conditions such as dementia is that the individual has never had personal experience of the decisions they are making in advance and they are unlikely to regain capacity ^20^.

**Figure 1.**
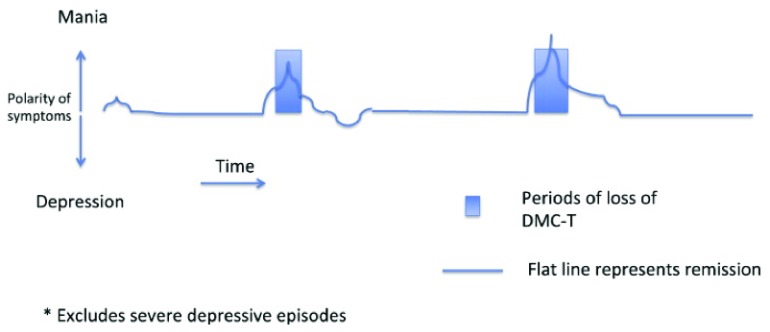
Fluctuating capacity (DMC-T) and remission in mania ^16^.

This is in stark contrast to the experience of someone with BP. Not everyone with BP will experience loss of capacity as defined by the MCA. However, loss of capacity for treatment decisions has been shown to be highly prevalent during manic episodes and can occur during a severe depressive episode ^7^. Given its fluctuating course, capacity can therefore be said to fluctuate in association with affect as depicted in Figure 1 ^16^. Loss of capacity is also common in other severe mental illnesses, although its fluctuating course is probably less predictable than that of BP ^21^.

Theoretically, this suggests that ADM would be well suited to BP. The first survey exploring interest in current MCA provision amongst people with bipolar in England and Wales strengthens this supposition ^22, 23^. The majority of respondents were in favour of using MCA provision to facilitate advance care planning for mental health crises. However use of the provisions for ADM under the MCA was low ^22^.

If ADM is to be expanded amongst the BP community, it is important to refine our understanding of the views of those with BP and how existing provision might be tailored to meet the needs of those using it. In particular, it is possible that people with BP may be practicing ADM without awareness of the MCA. In order to make this distinction, we have referred to “informal ADM” to mean advance plans that were created outside of the MCA framework for advance planning.

What’s more, as discussed above, those with BP may require compulsory inpatient admission and treatment and are therefore likely to want ADM provision to plan for this eventuality. One model of ADM which offers this is a self-binding directive (SBD) or Ulysses contract ^24, 25^. Gergel and Owen propose a model tailored for use by people with bipolar according to existing legal provision in England and Wales ^16^. An individual with bipolar works with a known clinician and family and/or friends to create a personalised capacity assessment. The information in the document would also inform the assessment for involuntary treatment with the aim that a person could be contained, according to their advance specifications, in hospital at an earlier stage in their episode of mania before significant damage occurs. Kane proposed an alternative account of this model which relies on a personalised notion of ‘risk to self’ rather than mental capacity ^26^. However, at the heart of both accounts is a willingness to engage with the idea of “self-binding” – the concept that the individual wants the contents of their plan to be respected even if they no longer agree to it when they are unwell.

In summary, the following has been discussed: BP is a severe mental illness in which capacity is likely to be lost during episodes of illness, ADM for such episodes is an autonomy promoting tool which has international support and is possible within multiple legal jurisdictions including England and Wales, the setting for this study. In addition, SBDs may be particularly pertinent for those with BP. Given these issues the challenge is to work towards a model of ADM which is satisfactory for people with BP. With this end in mind, the aims of this survey are outlined below.

## Aims

This study aimed to address the following objectives, with a focus on medical treatment:

1. To compare experiences of ADM with preferences for ADM amongst people with BP
2. To describe experiences of using ADM in crisis
3. To explore attitudes towards ADM including drivers for and barriers to ADM
4. To identify demographic and clinical variables that associate with interest in ADMs in general and SBDs in particular

## Methods

### Design

This study was an exploratory internet-based survey of people with BP in collaboration with Bipolar UK, the UK’s leading bipolar charity.

After reviewing the literature, a pilot questionnaire was designed addressing the study aims. This was reviewed by the research group which comprised two consultant psychiatrists, a research fellow with personal experience of BP, a specialist trainee in psychiatry and a specialist barrister in capacity law. The revised questionnaire was reviewed by two experienced BPUK employees before it was piloted on 13 people with BP and carers who provided written feedback. Follow-up interviews were conducted with a carer and a service-user to discuss the feedback in detail and trial possible alterations. The questionnaire was revised a second time by the research group. This resulted in the final questionnaire covering the following areas:

1. Experiences of ADM: use of ADM and experiences of utilising ADM during a crisis
2. Preferences for ADM: interest in ADM, content and production of ideal ADM document, attitudes toward self-binding
3. Attitudes towards ADM: drivers, barriers and concerns about ADM
4. History of mental illness: diagnosis, comorbidities, current care and history of BP episodes
5. Demographics: age, gender, ethnicity, education

A variety of single answer closed questions, multiple answer closed questions and open questions were utilised. Attitude and belief questions employed five-point Likert-type scales which have been shown to have adequate reliability and validity in a wide range of settings ^27^. Where possible, item-specific response scales were chosen rather than agree to disagree scales. This has been shown to reduce the degree of participant acquiescence and provide higher measurement quality ^28, 29^. “I don’t know” options were included in the majority of items. This was in response to feedback that the subject matter was too foreign for some pilots to provide a valid opinion.

The finalised questionnaire was uploaded on to “Bristol Online Surveys” ^30^. This is an online survey platform that is utilised by over 300 research organisations including 130 UK universities. A copy of this survey can be found on Harvard Dataverse ^31^. Please note that during the design and implementation of this survey, we used the term ‘advance care planning’ specifying if statutory or informal kinds were meant. For a single term, incorporating statutory and informal processes, we think “ADM” is preferable and use this for the purposes of the paper.

### Sample and distribution

The survey was distributed to the BPUK mailing list in October 2017. At time of distribution, this comprised 20,134 people who had registered their email and provided consent to be contacted by the charity.

The survey was open for 6 weeks. This was initially advertised by a dedicated email containing the URL to the online questionnaire with a description of the project and a request for participants. BPUK continued to promote the survey via social media, monthly newsletters, a reminder email and direct communication via support groups throughout the 6-week period to maximise response rate.

**Figure 2.**
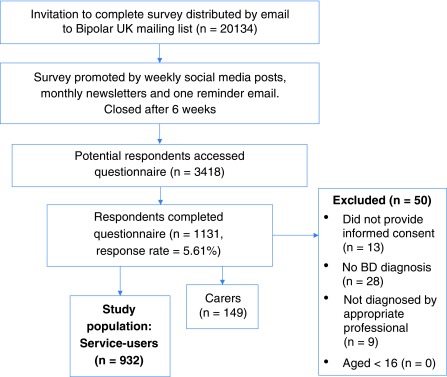
Flowchart describing survey distribution and generation of study population.

After 6 weeks 3418 people accessed the questionnaire. A total of 1131 completed the questionnaire or a parallel carer’s questionnaire. This constituted a response rate of 5.61%. There were 50 complete questionnaires excluded either due to lack of consent, no diagnosis of BP or lack of diagnosis by an appropriate professional. A total 149 respondents completed the carer’s questionnaire. This left 932 people with BP who met the inclusion criteria ( Figure 2).

### Analysis

Descriptive statistics were calculated using Microsoft Excel Professional Plus 2013® and Stata 15.0 ®.

To test for association, we generated the following binary outcomes of interest (dependent variables) from the raw data:

*Outcome of interest 1: endorsement of ADM*

This outcome was generated from the item “ *If you were supported by your mental health team to make an advance care plan, would you like to make one*?” (Supplementary Table 1 ^31^)

*Outcome of interest 2: interest in self-binding **AND** willingness to collaborate with a doctor*

This outcome was generated from two items. The first asked “ *Some people think a ‘self-binding statement’ is a good idea. This states that the person wants the contents of their advance care plan to be respected even if they no longer agree with it when they are unwell. Do you think this is a good idea?”* The second asked “ *Would you like to collaborate with your psychiatrist or GP to produce an advance care plan that they could sign showing they agree with the contents of the plan?”* (Supplementary Table 2 ^31^) This was designed as a proxy measure for interest in SBDs as these are essential components to the model as proposed by Gergel and Owen ^16^.

As there was an unexpectedly high proportion of respondents who indicated a preference for refusing all medication, we conducted a post-hoc analysis using a third outcome of interest:

*Outcome of interest 3: preference for refusing all medication*

This outcome was generated from the item “ *What information do you think an advance care plan should contain? – a) iii) refusing all medication?”*

Demographics, history of mental illness and attitudes towards involvement in decision making and trust in mental health practitioners were tested for association with the 3 outcomes of interest using χ ^2^ tests when all expected values were greater than 5 and Fisher’s exact tests when any expected values were less than 5 for categorical variables. T-tests and Mann-Whitney U tests were applied for normally distributed continuous variables and non-normally distributed continuous variables respectively. Using a cut-off of 0.05 significance, variables that were found to be significantly associated with the outcomes of interest were included in univariate logistic regression and multivariate regression corrected for potential confounders.

### Multiple comparisons

As our objectives are exploratory, we have presented findings with an initial threshold for statistical significance of <0.05. We subsequently used the Bonferroni-Holm method to correct for multiple comparisons. Results which cross this more rigorous threshold are indicated by ** and discussed in the text.

### Missing data

When presenting descriptive statistics, missing values have only been reported when greater than 5% total sample. When missing values are not reported, proportion of missing values is equal to the difference between the summed percentages and 100.

When performing logistic regression, missing data was assumed to be missing completely at random if missing values were less than 5% total sample. In these cases, a complete records analysis was conducted.

Two variables, “age” and “years since diagnosis”, had greater than 5% missing values. This data was assumed to be missing at random and so multiple imputation was applied when using these variables. Further details can be found in Supplementary Table 3 ^31, 32^.

### Ethics

Ethical approval was provided by the London – Surrey Borders Research Ethics Committee and Health Research Authority (REC reference number 17/LO/1071).

Informed consent was sought from potential participants prior to commencing the survey. Participants were given the opportunity to provide personal email addresses if they wanted to receive more information about the project in the future. These were uncoupled from the data prior to analysis to prevent loss of anonymity. No other identifying information was sought in the questionnaire.

## Results

### Sample demographics and disease characteristics

A total of 932 people with BP completed the survey and met the inclusion criteria. The sample had a mean age of 47.6 years and was predominantly female (71%), white British (87%) and had received a university level education (64%). Mean length of time since diagnosis was 12.7 years with 42% of respondents having received their diagnosis at least 10 years ago. In total, 61% had experience of hospitalisation and 56% were receiving secondary care or higher. A minority had experience of compulsory treatment (33%) ( Table 1).

**Table 1.** Sample demographics and disease characteristics.

Variable		n	% (n = 932)
**Age (years)** Mean = 47.6 SD = 12.6	< 30	85	9
	31 – 45	291	31
	46 – 60	357	38
	> 60 Missing	148 51	16 6
**Gender**	Male	251	27
	Female	665	71
	Other	10	1
**Ethnicity**	White British	808	87
	Other white	63	7
	Black British/ Caribbean/mixed	15	2
	Asian/mixed Asian	14	2
	Other	21	2
**Relationship** **status**	Married or cohabiting	501	54
	Other	421	45
**Education**	GCSE or lower	166	18
	A-levels	161	17
	University	598	64
**Years since** **diagnosis** Mean = 12.7 SD = 10.6	Less than 5	229	26
	5–10	258	28
	10 or more Missing	395 50	42 5
**Comorbidity**	Anxiety	444	48
	Post-traumatic stress disorder	109	12
	Panic disorder	100	11
	Emotionally unstable PD	99	11
	Other	343	37
**Level of care**	CMHT or similar	522	56
	Primary care	402	43
**Hospital** **admission**	Ever	573	61
	Never	348	37
**Compulsory** **treatment**	Ever	305	33
	Never	616	66

### Comparing experience of ADM with preferences for ADM

A total of 337 (36%) people responded positively to the item “ *have you ever written down or told someone about what you would like to happen to you or your affairs when you become unwell?*” Comparatively, 487 (52%) responded definitely yes and 337 (36%) responded probably yes to the question “ *If you were supported by your mental health team to make an advance care plan would you like to make one?”.* A smaller majority of people thought that a self-binding statement was either definitely a good idea (n = 356; 38%) or probably a good idea (n = 363; 36%).

**Figure 3.**
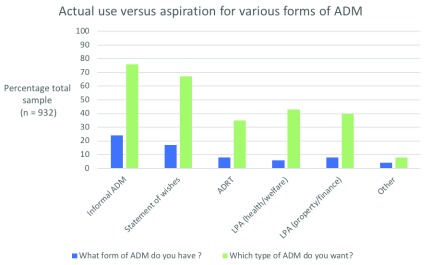
Bar chart comparing actual use with aspiration for various types of ADMs.

***Types of ADM.*** The majority of those with experience of ADM (66%) described their plans as “ *informal*” ( Figure 3). Of the MCA provisions for ADM, advance statements of wishes and preferences were most common (17% of total sample), while ADRTs and LPAs for health and welfare and property and finance were less than half as prevalent (6% and 8% respectively). A significant proportion of all forms of ADM were communicated verbally (42%; n = 274). A total of 129 (38%) respondents reported owning one or more legally binding form of ADM, 82 (24%) of which had been communicated in writing.

Most people stated a preference for non-legally binding ADM. In total, 771 (76%) indicated they would make informal ADs and 627 (67%) advance statement of wishes and preferences. Fewer respondents expressed an interest in making ADRTs (35%), LPA health and welfare (43%) or LPA property and finance (40%) ( Figure 3).

In all types of ADM there was a mismatch between aspiration and actuality with higher percentages wanting to engage in ADM than had experience of ADM.

***Content of Advance Decisions.*** Respondents indicated that “ *contact details of who to notify*” and *“who makes decisions if you are unable to make decisions for yourself”* were the most frequent pieces of information contained in their AD (71% and 68%, respectively). A total of 40% requested specific medication, 35% refused specific medications and 33% requested hospitalisation; 10 respondents (3%) had attempted to refuse all medication ( Table 2).

**Table 2.** Table comparing the actual content of people’s ADs (coloured blue) with the aspirational content (coloured green). Aspirational content is divided into two samples (1) the subsample with ADs and (2) the total sample.

Content of ADs	*What information does* *your AD contain?*		*What should an AD contain?*			
	**People with ADs**		**People with ADs**		**Total sample**	
	**n**	**% (n = 337)**	**n**	**% (n = 337)**	**n**	**% (n=932)**
Requesting specific medications	134	40	237	70	639	69
Refusing specific medications	118	35	245	73	636	68
Refusing all medications	10	3	78	23	190	20
Requesting ECT	17	5	95	28	241	26
Refusing ECT	104	31	229	70	585	63
Requesting hospitalisation	110	33	220	65	631	68
Where to be treated	108	32	246	73	649	70
Where not to be treated	102	30	212	63	581	62
Description of behaviour or speech used when in a crisis	154	46	253	75	642	69
Planning your discharge	33	10	211	63	568	61
Who makes decisions if you are unable to make decisions for yourself	230	68	313	90	827	89
Who takes responsibility for your finances	159	47	258	77	720	77
Contact details of who to notify	238	71	294	87	806	87
Who not to notify	80	24	182	54	485	52

A similar pattern is presented when participants were asked what information ADs should relate to, with “ *contact details of who to notify*” and *“who makes decisions if you are unable to make decisions for yourself”* also ** being the most frequently selected (87% and 89% of the total sample, respectively). An equivalent proportion of the total sample wanted to request specific medications (69%) as well as refuse specific medication (68%) and request hospitalisation (68%). A substantial number of people indicated a wish to refuse all medication (20%) ( Table 2).

For all content there was a mismatch between aspiration and actuality with respondents indicating a preference for ADs that cover a broad range of options. The content aspiration in the subsample with experience of ADM was similar to the total sample ( Table 2).

***Support and storage.*** Respondents most frequently produced their ADM with the support of family/friends (42%) or a CPN/care co-ordinator (37%) ( Table 3a); 14% had worked with a psychiatrist and 7% with a GP. A minority of ADMs was present within psychiatric (36%) or GP (21%) notes. In contrast, 70% of participants indicated a preference to collaborate with a psychiatrist and 55% with a GP. Family and friends remained the most popular group with 79% preferring their support. Most respondents reported a preference for ADMs to be kept within psychiatric or GP notes (79% and 83%) as well as with family and friends (70%) ( Table 3b).

**Table 3.** Table comparing (a) who was actually involved in producing the advance decision making (ADM) (blue) with aspiration for who should be involved (green) and (b) where ADM is actually stored (blue) with aspiration for where it should be stored (green). Aspirations are divided into two samples (1) the subsample with ADs and (2) the total sample.

a) Support-giver	*Who else was involved in* *producing your AD?*		*Who else should be involved in* *producing an AD?*			
	People with ADs		People with ADs		Total sample	
	n	% (n = 337)	n	% (n=337)	n	% (n=932)
Psychiatrist	48	14	232	69	650	70
GP	24	7	181	54	517	55
Care co-ordinator/CPN	124	37	209	62	505	54
Family/friends	141	42	265	79	736	79
Lawyer	16	5	79	23	218	23
Alone	57	17	18	5	36	4
Other	40	12	78	23	191	20
**b) Storage method**	***Where is your AD stored?***		***Where should an AD be stored?***			
Psychiatric notes	122	36	283	84	737	79
GP notes	70	21	283	84	775	83
At home	111	33	184	55	385	41
Family/friends	116	34	266	79	657	70
Other	43	13	37	11	85	9
I don’t know	38	11	5	1	24	3

Again, there was an actuality/aspiration mismatch for support and storage with higher percentages wanting support and storage than had it. The support and storage aspiration in the subsample with ADM was similar to the total sample.

### Making ADs and their use in crisis

In total, 45% of respondents with ADs were satisfied with the contents of their plan, 49% felt more in control because of their plan and 51% felt more involved in decisions about their healthcare. Despite this, a minority (26%) of respondents had experience of their ADs being used in a crisis ( Table 4).

**Table 4.** Experiences of making an advance decision (AD).

Question/statement	Collapsed Likert scale	n	% (n = 337)
*How satisfied are you with* *the contents of your AD?*	Satisfied	152	45
	Neutral or Unsatisfied	135	40
	I don’t know	45	13
*I feel more in control as a* *result of my AD*	Agree	162	48
	Neutral or disagree	131	39
	I don’t know	23	7
*I feel more involved in* *decisions about my* *healthcare as a result of* *my AD*	Agree	173	51
	Neutral or disagree	122	36
	I don’t know	19	6
	Missing	23	7
*How many times has your* *AD been used in a crisis?*	Never	207	61
	Ever	88	26
	I don’t know if it’s been used	25	7

Of these respondents (n = 88), 50% felt their AD was highly or completely respected by mental health staff, 60% felt they had recovered faster as a result of their AD and 60% had a better experience of health services; 55% were happy with how their AD had been used in general. ADs were reported to be more effective in reducing harm to relationships (44% very or extremely helpful) and physical harm (34%) than reducing overspending (23% very or extremely helpful) ( Table 5).

**Table 5.** Experiences using an advance decision (AD) in a crisis.

Question	Collapsed Likert scales	n	% (n=88)
*To what extent did* *mental health staff* *respect the contents of* *your AD?*	Highly - completely	44	50
	Slightly - moderately	31	35
	Not at all	12	14
*What effect did your AD* *have on your recovery* *from your crisis?*	Faster	53	60
	No effect	26	32
	Slower	2	2
	I don’t know	7	8
*What effect did* *your AD have on* *your experience of* *treatment?*	Better	53	60
	No effect	29	33
	Worse	6	7
	I don’t know	4	5
*How helpful was your* *AD in reducing physical* *harm?*	Very - extremely	30	34
	Slightly - moderately	25	28
	Not at all	18	20
	Missing	15	17
*How helpful was your* *AD in reducing harm to* *relationships?*	Very - extremely	39	44
	Slightly - moderately	26	30
	Not at all	14	16
	Missing	9	10
*How helpful was* *your AD in reducing* *overspending?*	Very - extremely	20	23
	Slightly - moderately	35	40
	Not at all helpful	21	24
	Missing	12	14
*How happy are you* *with how your AD has* *been used in general?*	Happy	48	55
	Neutral or unhappy	39	44

### Drivers for and barriers to ADM

The most frequently selected reason to make an AD was “ *I would be more involved in decisions about my mental health*” (84% of respondents). This was followed by “ *reduce pressure on family and friends to make decisions on my behalf*”, “ *improve my experience of being treated by mental health services*” and “ *I would be more in control of my illness*” (74% and 70%, respectively) ( Table 6).

**Table 6.** Attitudes towards drivers for ADM.

Which of the following do you think are the most important reasons to make an advance decision?	n	% (n = 932)
*I would be more involved in decisions* *about my mental health*	787	84
*Reduce pressure on family and friends to* *make decisions on my behalf*	690	74
*Improve my experience of being treated* *by mental health services*	655	70
*I would be more in control of my illness*	643	69
*Reduce harm to relationships*	557	60
*Faster recovery from an episode of mania* *or depression*	545	59
*Reduce harm to myself during an episode*	509	55
*Reduce risk of overspending*	485	52
*Reduce harm to property*	261	28
*I don't think there are any significant* *benefits*	17	2

In terms of barriers, a minority (25%) agreed with the statement “ *I don’t see the point because Advance Decisions to Refuse Treatment can be overruled by the Mental Health Act”*, ** despite a majority (71%) indicating that it is very or extremely important that ADM should be legally binding. 47% indicated that they thought it would be difficult to make an AD while only 19% felt it would be too distressing to do so. A significant minority agreed with the statement “ *I don’t understand enough about advance care planning*” (47%) although most respondents believed that they understood their condition well enough to make an AD (72%) ( Table 7).

**Table 7.** Attitudes towards potential barriers to ADM.

Collapsed Likert scales	Agree	Neutral	Disagree
n = 932	n (%)	n (%)	n (%)
*I don’t understand enough about ADM*	434 (47)	202 (22)	275 (30)
*I don’t understand my condition well enough to* *make an AD*	104 (11)	134 (14)	673 (72)
*I don’t see the point because Advance Decisions to* *Refuse Treatment can be overruled by the Mental* *Health Act*	233 (25)	317 (34)	360 (39)
*Mental health workers have more than enough time* *to help with ADM*	141 (15)	183 (20)	599 (64)
*It would be too distressing to think about times when* *I have been unwell during the process of making an* *AD*	173 (19)	207 (22)	531 (57)
*I don’t think mental health teams would be able to* *access an AD when they need it*	338 (36)	303 (33)	275 (30)
*I don’t think mental health practitioners would* *respect an AD*	313 (34)	318 (34)	284 (30)
	**Easy**	**Neutral**	**Difficult**
*How easy do you think it is to make an AD?*	198 (21)	298 (32)	433 (47)
	**Very/extremely**	**Slight/moderately**	**Not at all**
*How important is it to you that an AD should be* *“legally binding”?*	665 (71)	227 (24)	32 (3)

### Demographic and clinical variables associated with interest in ADM and SBDs, and preference for refusing all medication

Of the respondents, 824 (88%) registered an interest in ADM (Supplementary Table 1 ^31^) and 641 (69%) responded positively to the self-binding concept and to collaboration with a doctor (Supplementary Table 2 ^31^).

Table 8 shows associations with ADM in general. Of note there was no association with gender, ethnicity or education. There was a statistically significant association at the 0.05 threshold between interest in ADM and a younger age, shorter time since diagnosis, higher level of care, ever experiencing mania and a greater trust in healthcare professionals (Supplementary Table 4). After controlling for key demographic variables, younger age, history of manic episodes and trust in healthcare professionals remain significantly associated. Only the association with younger age remained significant after correction for multiple comparisons.

**Table 8.** Interest in any ADM: univariate and corrected associations. Univariate regression of age and years since diagnosis, and all multivariate regression were conducted using multiple imputation.

		Univariate regression Odds ratio (95% CIs)	Model corrected for age, gender, ethnicity, education and comorbidity Odds ratio (95% CIs)
Age*		**0.97 (0.95 – 0.98)** p < 0.0005 **	0.97 (0.95 – 0.98) p < 0.0005 **
		n = 922	n = 896
**Years since diagnosis**		**0.97 (0.96 – 0.99)** **p = 0.002**	0.99 (0.96 – 1.01) p = 0.222
		n = 926	n = 896
**Level of care**	GP	1.00	1.00
	CMHT or higher	**1.62 (1.07 – 2.46) p = 0.024**	1.49 (0.96 – 2.31) p = 0.074
		n = 916	n = 894
**Manic episode**	0	1.00	1.00
	1–4	**3.02 (1.13 – 8.12)** **p = 0.028**	**3.34 (1.24 – 9.35)** **p = 0.021**
	5 or more	**3.36 (1.26 – 8.99)** **p = 0.016**	**4.55 (1.62 – 12.81)** **p = 0.004**
	Don’t know	1.85 (0.61 – 5.66) p = 0.279	2.03 (0.64 – 6.58) p = 0.232
		n = 913	n = 891
**Trust in healthcare** **professionals**	Agree	1.00	1.00
	Neutral	0.595 (0.35 – 1.02) p = 0.058	0.64 (0.37 – 1.11) p = 0.116
	Disagree	**0.48 (0.29 – 0.79)** **p = 0.004**	**0.49 (0.29 – 0.83)** **p = 0.008**
		n = 916	n = 896

**indicates statistical significance after Bonferroni-Holm correction for multiple comparisons.

Table 9 shows associations with interest in self-binding with a doctor’s involvement. There was no association with age, ethnicity, educational level or gender (Supplementary Table 4 ^31^). Interest in self-binding with doctor’s involvement was significantly associated with experience of compulsory treatment, detention by police and greater trust in healthcare professionals. The association with all three variables remained significant after controlling for key demographics. Only the association with trust in healthcare professionals was significant after Bonferroni-Holm correction.

**Table 9.** Interest in SBD: univariate and corrected associations. Multivariate regression was conducted using multiple imputation.

		Univariate regression Odds ratio (95% CIs)	Model controlling for age, gender, ethnicity, education and comorbidity Odds ratio (95% CIs)
**Involuntary detention**	Never	1.00	1.00
	Ever	**1.37 (1.01 – 1.86)** **p = 0.042**	**1.55 (1.13 – 2.13)** **p = 0.007**
		n = 918	n = 894
**Detention by police**	Never	1.00	1.00
	Ever	**1.42 (1.02 – 2.00)** **p = 0.040**	**1.45 (1.02 – 2.06)** **p = 0.036**
		n = 908	n = 898
**Trust in healthcare** **professionals**	Agree	1.00	1.00
	Neutral	**0.57 (0.40 – 0.81)** **p = 0.002**	**0.58 (0.40 – 0.83)** **p = 0.003**
	Disagree	**0.43 (0.30 – 0.81)** **p < 0.0005** ^**^	**0.43 (0.31 – 0.61)** **p < 0.0005** ^**^
		n = 921	n = 901

**indicates statistical significance after Bonferroni-Holm correction for multiple comparisons.

To explore the possibility of a confounding effect between compulsory treatment and detention by police, we performed further multivariate regression analysis. There was moderate collinearity between these variables (r = 0.39, p < 0.00005). When controlling for detention by police, the association between a history of involuntary detention and interest in self-binding with clinician involvement was no longer significant (OR 1.22, CIs 0.87-1.70, p = 0.242). When controlling for experience of involuntary detention, the association with detention by police also became non-significant (OR 1.32, CIs 0.92-1.91, p = 0.134). There was no significant interaction between these two variables.

A preference for refusing all medications as part of ADM was associated with younger age, fewer years since diagnosis, never experiencing hospitalisation, female gender and a lack of trust in healthcare professionals (Supplementary Table 5 ^31^). When controlling for confounders, only younger age, female gender and lack of trust in healthcare professionals remained significant. Younger age and lack of trust in healthcare professionals remained significant after correction for multiple comparisons ( Table 10). There was no association between preference for refusing all medication and an interest in ADM (χ ^2^ = 0.45, p = 0.504), or interest in self-binding with doctor’s involvement (χ ^2^ = 0.91, p = 0.341).

**Table 10.** Preference for advance refusal of all medication: univariate and corrected associations. Univariate regression of age and years since diagnosis, and all multivariate regression was conducted using multiple imputation.

		Univariate regression Odds ratio (95% CIs)	Model controlling for age, gender, ethnicity, education and comorbidity Odds ratio (95% CIs)
**Age**		**0.97 (0.95 – 0.98)** **p < 0.0005** ^**^	**0.97 (0.96 – 0.98)** **p < 0.0005** ^**^
		n = 924	n = 897
**Years since diagnosis**		**0.97 (0.95 – 0.99)** **p < 0.0005** ^**^	0.99 (0.97 – 1.01) p = 0.322
		n = 928	n = 897
**Hospitalisation**	Never	1.00	1.00
	Ever	**0.71 (0.44 – 0.98)** **p = 0.038**	0.75 (0.54 – 1.06) p = 0.103
		n = 921	n = 891
**Gender**	Male	1.00	1.00
	Female	**1.75 (1.18 – 2.61)** **p = 0.005**	**1.58 (1.05 – 2.38)** **p = 0.029**
	Other	2.56 (0.63 – 10.36) p = 0.188	2.70 (0.64 – 11.40) p = 0.177
		n = 926	n = 897
**Trust in healthcare** **professionals**	Agree	1.00	1.00
	Neutral	1.27 (0.84 – 1.94) p = 0.260	1.22 (0.79 – 1.89) p = 0.366
	Disagree	**2.35 (1.61 – 3.41)** **p < 0.0005** ^**^	**2.08 (1.41 – 3.08)** **p < 0.0005** ^**^
		n = 923	** n = 897**

**indicates statistical significance after correction for multiple comparisons.

## Discussion

This study is the largest survey of attitudes towards ADM amongst people with BP, who have traditionally been a hard to reach group. It provides an important step forward in understanding experiences and attitudes towards ADM in this group. Furthermore, it offers insight into how people with BP would like ADM provision to be shaped. In a field where the results of empirical studies are conflicting but the intervention seemingly has high-levels of support among service-users and clinicians alike ^33– 35^, this understanding is vital in developing accurately tailored interventions.

Most strikingly, a large majority of this study’s sample have indicated that they would like to plan their care in advance of losing capacity (88%) but most have not done so (64%). Of those who have engaged in ADM, a minority have experience of their plans being used in crisis. When they are used, respondents report generally positive experiences, with 50% feeling that their ADs were respected either highly or completely, 60% indicating that they recovered faster and 60% experiencing better treatment because of their ADs. There is also a notable interest in SBDs, with 69% expressing an interest in self-binding as well as a willingness to collaborate with doctors.

### Limitations

There are several important limitations to this study. This is a predominantly white (94%), female (71%), well-educated sample (64% university educated) which is not representative of the wider population of people with bipolar in the UK (88.8% white, 48.9% female, and 36% university educated) ^36^. The use of BPUK mailing list as the sampling frame and a low response rate introduce further selection bias. People with a prior awareness or interest in ADM are likely to be over-represented. It is therefore difficult to generalise these findings and the study should be seen as exploratory.

Nonetheless, representative opinions of people with severe mental illness are hard to access and the only comparable study performed in the UK reported a sample size of 544 with an unknown response rate due to the sampling methodology employed ^22^. This study’s larger sample size and transparent methodology thus helps take the literature a step forward. The use of other methodologies to sample the attitudes of hard-to-reach groups such as respondent-driven sampling may be useful ^37^. While in order to understand the generalisability of these findings, a simplified version of the survey should be conducted using a more representative sampling technique such as convenience sampling through outpatient clinics or systematic sampling through the use of a patient research database such as the Clinical Record Interactive Search system ^38^.

It is notable that only a minority of our sample have experience of involuntary detention (33%). Arguably, this could suggest that the majority of this sample have a less severe manifestation of BP, are therefore less likely to lose capacity during episodes, meaning ADM may not be relevant for these respondents. However, the majority do have experience of hospitalisation which is an important indicator of severity. In addition, during an inpatient admission which coercion, leverage and treatment pressures can be present in the absence of legally mandated detention ^39, 40^. What is more, there was no association between experience of compulsory treatment and interest in ADM, suggesting that ADM does appeal to people who have not experienced involuntary detention.

We utilised a proxy-measure for interest in SBDs by combining interest in self-binding statements with a willingness to collaborate with doctors. This was because we decided that attempting to explain SBDs in a survey was likely to generate confusion. This means our results cannot be interpreted as a direct endorsement for SBDs. We have conducted focus groups in which SBDs were discussed as part of a parallel study which will help to triangulate these findings.

A quantitative survey was employed to explore and characterise a large number of people’s views. The use of closed response options, however, limits the depth of opinions and experiences reported. What’s more it is possible that some participants misunderstood some of the complex concepts contained within the questionnaire. Our focus on ADM in relation to medical treatment may have added to this potential misunderstanding. ADM can relate to financial or work matters and it is possible that some of our respondents may have been referring to these in some of their responses. Further structured qualitative analysis of the free text boxes included in the survey will be reported in a future publication, helping to disambiguate this. While further research is needed to explore to what extent these findings compare to other forms of ADM.

### Consistency with previous studies

Some of our results replicate findings reported by Morriss *et al.* in a survey of the utilisation of the MCA by people with BP ^22^. Use of advance statement of wishes and feelings and LPAs (property/finance) were somewhat more prevalent among our study’s sample (17% and 8%, respectively) compared to Morriss *et al.* (11.4% and 3.9%, respectively). It is possible that use of these provisions has increased over time. Use of ADRTs and LPAs (health/welfare) was more equivalent, 8% and 6% compared to 9.9% and 5.7%, respectively, obtained by Morriss *et al.* In terms of interest, 74.1% “believed making plans about their personal welfare if they lost capacity to be very important” compared to 88% of our sample that expressed interest in ADM ^22^.

Comparable findings have been reported in wider populations of people with severe mental illness rather than just BP. Swanson and colleagues found that 4–13% of people with mental illness in a large multicentre cohort from the US had psychiatric advance directives while 66–77% of this sample wanted to make one ^41^.

### Therapeutic potential of ADM in crisis care and beyond

In this study, of the minority of respondents who did have experience of using ADM in crisis, 50% indicated their plan was highly or completely respected and 60% felt they had recovered faster and had a better experience of being treated by mental health services. This builds on Srebnik and Russo’s finding in the U.S. that two thirds of treatment decisions were consistent with participants’ psychiatric advance directives ^42^. This is preliminary evidence that ADM produced outside of an “experimental” setting *can* be used in crisis with therapeutic effect and that they tend to be respected when they are used, although it is worth noting that significant proportions of our sample did not report these positive experiences.

Usability and respect have previously been raised as critical issues facing the implementation of ADM ^43^. However, as previously discussed, in England and Wales the current legal framework may act as a barrier to ADM implementation for those with mental illness in that there is no MHA provision for formal recognition of ADM; Morriss *et al.* found only 12.5% of people with ADRTs had their plans respected when detained under the MHA ^22^.

Half of those with ADs felt more in control because of their plan and felt more involved in decisions about their healthcare. Wauchope *et al.* reported similar positive changes in feelings of independence, control and motivation in the majority of people who were assisted in making ADM ^44^. This suggests there are benefits of ADM beyond its role in crisis and that even if specific legal provision for ADM for people with mental illness is not available in the near future, ADM is still experienced as worthwhile by a large proportion of people with mental illness.

### Desire for mental health service involvement

The limited involvement of psychiatrists and GPs in the production and storage of ADs is striking. Only 21% of ADs were made with the help of either a psychiatrist or GP and only 36% were present in psychiatric notes. This suggests that, for most people, ADM is practised outside of the medical sphere and for many it is a “do-it-yourself” exercise with family and friends alone. Morriss *et al.* report similar findings, with only 48% of ADRTs having being produced with the help of mental health professionals and 48% present in mental health notes ^22^.

The extent to which mental health services currently engage with ADM is likely to be a key factor, although little is known about current practice in the UK at NHS Trust level. Bartlett *et al.* reported that psychiatrists *“would discuss more if patients requested it”* ^23^. Internationally, a degree of ambivalence among mental health professionals has also been described. Elbogen reported that only 47% of psychiatrists, psychologists and social workers believed that ADM “ *would be helpful to consumers*” ^45^ and only 28% of Scottish psychiatrists thought advance directives were needed in Atkinson and colleagues’ survey ^46^. Swanson demonstrates the wide gap between clinicians and service-users attitudes, with 78.43% of service users believing that “advance instruction will help people…stay well” compared to only 43.9% clinicians ^47^.

Some have suggested that this is the way ADM should be conducted: for ADM to truly enhance autonomy, they should be completed without the involvement of mental health professionals ^41, 46, 48– 51^. In fact, Henderson *et al.* reported a preference among US veterans for plans to be produced outside of a mental health setting and the need for “nonpartisan assistance” ^52^. What´s more, Ruchlewska *et al.* found that “crisis plans” facilitated by non-clinicians performed better in a “crisis plan quality checklist” than crisis plans completed with clinicians, although there was no difference in performance clinically ^34, 53^.

In our sample there was a clear preference for doctors to be involved and for plans to be present in medical notes, and interest in ADM was associated with *increased* trust in mental health professionals. Similar attitudes have been reported among service users previously as well as other key stakeholders and this collaborative approach has been employed by several clinical trials of ADM-type interventions ^34, 43, 54, 55^. This would suggest that our sample favours a “therapeutic alliance” approach with clinical “buy-in” rather than an anti-medical model ^43^.

These inconsistent findings in the literature may be due to sampling biases, as well as cultural differences between different countries. However, it also highlights the breadth of attitudes present and the challenges in identifying the “right” way to implement such an intervention.

There are several potential consequences to mental health services failing to take an active role in ADM. Firstly, our findings suggest that ADM is currently ineffective for many people, with 61% of ADs never having been used in a crisis. This is perhaps unsurprising given 64% of plans are absent from mental health notes. Other UK studies also suggest that only a minority of ADM that are created are recorded in written form ^22, 23^. This is of concern given that in England and Wales at least, multiple agencies may be involved in a crisis: mental health services, friends and family, social services and the police. When individuals from these professional groups were asked about ADM, lack of accessibility to electronic and paper documents across services and localities was seen as a key barrier to implementation (Stephenson *et al.*, personal communication).

Secondly, without mental health service involvement, the chance of ADM being a source of conflict and disappointment is higher. This is most evident in the finding that 20% of this sample expressed a wish to refuse all medication. While this may be achievable for some, it is possible that treatment would be enforced despite their wishes if they were detained under the MHA. Service-users refusing all medication has previously been described as the biggest concern about ADM among healthcare professionals and may contribute to the degree of ambivalence described above ^45, 56, 57^. We have found that preference to refuse all medication associates with younger age, which may suggest a need for targeted engagement of younger patients to help avoid conflict and disappointment later. However, the strong association with participants who have less trust in healthcare professionals suggests this may be a challenging group to engage.

Thirdly, our sample show high interest in SBDs, particularly amongst those with more experience of compulsory treatment. These are types of ADM that will require collaboration with mental health services to work as they involve requests, rather than refusals, of treatment, often at earlier stages than would otherwise happen under the MHA.

Finally, as awareness of ADM increases among people with BP and other mental illnesses, the need for clinicians to engage at the planning stage may become increasingly evident, as clinicians will be expected to respond to questions about ADM that have clinical implications ^58^.

### Tailoring advance decision making for bipolar

***Medication refusal.*** The finding that 20% of our sample would like to refuse all medication is substantially higher than findings in equivalent surveys of people with severe mental illness, not specifically BP, with figures typically between 0–5% ^41, 59^. Some of our findings are more consistent with samples that are not BP specific. Refusals of ECT, for example, range from 42% ^59^ to 72% ^58^ while this would be included by 70% of our sample who already had ADs and 63% of the entire sample. However, others appear to differ more significantly. A total of 96% of Scottish advance statements reviewed by Reilly *et al.* included a specific medication refusal, while only 35% of ADs in our sample included this information and 68% of the total sample would include this. Conversely, only 45% of the sample assessed by Reilly *et al.* requested medication, while 68% of our sample would like to include this ^59^.

These differences may be due to sampling differences. However, this is the first study to explore these issues among a BP-specific population. It is therefore possible that there are important differences in attitudes between people with different diagnoses and that this provides evidence against a “one size fits all” approach.

***Advance requests for coercive treatment: self-binding directives.*** Difference by diagnosis may also be true for the degree of interest in SBDs. A study of 104 people with schizophrenia found that the majority believed that people should be able to revoke an AD even when lacking capacity for treatment decisions ^47^. This attitude is in direct opposition to the capacity based model proposed by Gergel and Owen in the bipolar context which stipulates that the service-user receives compulsory treatment as outlined in their SBD when lacking capacity for treatment decisions in the knowledge that they may resist this treatment when unwell ^16^. The majority of bipolar participants in this survey supported the idea that contents of their advance care plan should be respected even if they no longer agree with it when they are unwell.

Our findings also provide an interesting comparison to some of the clinical trials of ADM-type interventions. In our sample, 68% wanted to be able to request hospitalisation as part of an AD. This is in contrast to several of the trials which included reduction of hospitalisation rate as an outcome measure, all of which failed to demonstrate any effect ^33, 35, 57^. This helps to illustrate a key debate around the appropriate outcome measures for ADM: is reduction in hospitalisation the critical test of their value? The fact that the five most frequently selected reasons to engage in ADM were related to self-determination and relationships suggest that our sample supports a wide view on the appropriate outcome measures for ADM (see Table 6).

### Inconsistent attitudes towards legal status

Respondents expressed a preference for non-legally binding forms of ADM which included information about refusing medication or substitute decision makers over legally binding forms that could communicate the same information such as ADRTs and LPAs. This is despite 71% of respondents indicating that they thought it was either very or extremely important that an AD should be “legally binding”. This apparent contradiction may be due to a lack of understanding both of the terminology but also the legal status of different options available. This is supported by the fact that 47% responded that they *“did not understand ADM well enough”.* Low levels of awareness of the MCA has previously been reported among people with BP ^22^ while limited awareness of ADM in the among people with mental illness including dementia has also been identified as a key barrier to its implementation ^60, 61^.

Participants’ attitudes in principle may also differ to their attitudes in practice. It is possible that participants believe that it is extremely important that ADMs should be legally binding when asked in general terms. However, when given the choice of making their own plan and invited to consider the practical aspects of making a plan, the idea of “binding” themselves and others to that plan may seem less appealing. This effect has been described in people approaching end-of-life care who may feel “intimidated” by something perceived to be legally binding in practice ^62^.

However, this may also reflect a lack of interest in the legally binding provisions currently available in England and Wales. Much of the preferred content for ADM in our sample, including requesting medication and hospitalisation, falls outside of the legally binding provisions under the MCA. ADRTs and LPAs may therefore seem too limited and restrictive despite their legally binding status.

A further possibility is that respondents may prefer the best of both worlds; provision with sufficient legal force for their wishes to be taken seriously yet with sufficient flexibility which accommodates the difficulties of predicting future situations.

### Associations with interest in ADM and SBDs

Focus groups exploring psychiatrists’ attitudes towards ADM and SBDs (Stephenson *et al.* personal communication) have elicited assumptions that only a certain “type” of patient will have the resources to engage in ADM. Although there may be subtle associations that are not apparent due to sample size or a non-representative sample, it is notable that there was no evidence that interest in ADM nor SBD with doctors’ involvement are associated with education, ethnicity or gender. This is replicated in the survey by Morriss *et al.*, which identified a positive association between knowledge of the MCA and higher educational level but not use of the MCA ^22^. These assumptions are likely to contribute to the lack of involvement among mental health staff who *“would discuss more if patients requested it”* ^23^.

In contrast, in their survey of interest in Psychiatric Advance Directives among people with severe mental illness, Swanson *et al.* demonstrated a positive association with women, non-white respondents, lower educational level, recent contact with police, high pressure to take medications and low sense of personal autonomy. This was interpreted as an attempt by a disenfranchised and disillusioned population to regain control ^41^.

The finding that interest in SBD and ADM are significantly associated with increased trust in healthcare professionals may be evidence against this hypothesis among our sample. Indeed, it is likely that people with less trust in their mental health services do not trust their services to enact an AD and therefore do not express an interest. Similar attitudes were elicited in a qualitative study by Wauchope *et al.* exploring the process of developing ADM for people with severe mental illness, observing that participants with negative experiences of mental health services were more likely to be suspicious of the process ^44^.

The variables associated with interest in ADM and SBD otherwise differ. This suggests that the group of people interested in SBD represent a distinct sub-group to those that are interested in ADM in general. Most importantly, interest in SBD was associated with experience of compulsory treatment. Coercive treatment is central to the SBD model, where the service user seeks to have a voice in the kind of coercive treatment that is needed for them based on their past experience of illness episodes ^16^. This positive association is therefore evidence that the concept has been understood and that there is interest among the people for whom it is most relevant. Meanwhile, the finding that age is inversely associated with interest in ADM is in keeping with a generational effect namely that younger people are more likely to assume an active role in healthcare decisions and less likely to assume medical paternalism ^63^.

### Future directions

These results demonstrate sizeable appetite for ADM among a large group of people with BP and a desire for input from mental health services in creating and implementing ADM for therapeutic effect. However, despite this appetite, ADM remains uncommon in clinical practice. Translating this enthusiasm into clinical practice therefore presents a major challenge.

Firstly, it is difficult to envisage mental health trusts prioritising ADM while it remains an afterthought within mental health legislation in England & Wales. However, this may change. The recently published report from the Independent Review of the Mental Health Act, to which data from this survey contributed, has included provision for ADM as one of its key recommendations. This presents an exciting opportunity to think through and promote ADM within mental health with renewed vigour and may lead to new legislation ^64^.

Legislation alone is unlikely to be successful. Our findings, and others, suggest a lack of engagement among mental health staff that is likely to stem from resource and training issues, priorities within mental health trusts and assumptions around who may be interested in and able to use ADM ^23, 65^.

To enable service users and clinicians to harness the potential of ADM top down facilitation and bottom up pressure is required. Mental health services need appropriate resources and the development of clinically feasible ADM focussed care pathways, resources and guidelines. In addition, ongoing advocacy and awareness raising from third sector organisations such as BP UK can help to raise knowledge of ADM amongst the service user community and encourage informed requests for ADM.

Finally, while it is promising that a large proportion of this sample endorses SBDs, this study’s methodology does not allow these attitudes to be explored in depth. There are also several key stakeholders whose input and buy-in is essential for the model to be implemented successfully. We have therefore conducted focus groups with service-users, mental health practitioners and lawyers exploring some of the key ethical and practical aspects of SBDs (Stephenson Unpublished). Building on these findings, we will trial one model of clinician/service-user co-produced SBDs for people with BP which will commence in 2019.

## Conclusions

This study explores the attitudes and experiences of people with BP towards ADM in the UK. It has demonstrated that ADM is uncommonly practised despite substantial interest. The results suggest services users with BP want to use ADM and they want collaborative input from mental health services in doing so, although the low response rate mean these results should be generalised with caution. We hope that the results of this study combined with the opportunities provided by the Independent Review of the Mental Health Act, will help to translate this interest into action and enable people with BP to extend their autonomy to situations in which their autonomy is threatened.

## Data availability

### Underlying data

Due to risk of de-anonymisation, the raw underlying data has not been made freely available. If researchers or referees would like access to the data for re-analysis, please contact the corresponding author (L.A.S.) by email at lucy.a.stephenson@kcl.ac.uk.

### Extended data

Harvard Dataverse: “A survey of experiences of and attitudes to advance decision making amongst people with bipolar: questionnaire and supplementary information”, https://doi.org/10.7910/DVN/WHUYQR ^31^.

The original questionnaire and a document containing Supplementary Tables 1–5 are included as extended data.

Data are available under the terms of the Creative Commons Zero “No rights reserved” data waiver (CC0 1.0 Public domain dedication).
