# Supplementary material for: “Why have I not been told about this?”: a survey of experiences of and attitudes to advance decision-making amongst people with bipolar
Source: Wellcome Open Res. 2019 Apr 23;4:16. Originally published 2019 Jan 29. [Version 2] doi: 10.12688/wellcomeopenres.14989.2 (PMC6492047; doi:10.12688/wellcomeopenres.14989.2)
Supplement: Peer review history

# Peer review: Reviewer response for version 2

**DOI:** 10.21956/wellcomeopenres.16626.r35357

**Published:** 2019-06-12

**Authors:** Nick Craddock

**Affiliations:** 1 Institute of Psychological Medicine & Clinical Neurosciences, Cardiff University, Cardiff, UK

The authors have addressed the concerns I raised in my first review by moderating their claims and providing a much improved discussion of the limitations of their study. It is now much improved and is a useful contribution to the literature that it can be hoped will stimulate future systematic studies of advanced decision making in people with bipolar disorder.

I have read this submission. I believe that I have an appropriate level of expertise to confirm that it is of an acceptable scientific standard.

# Peer review: Reviewer response for version 2

**DOI:** 10.21956/wellcomeopenres.16626.r35355

**Published:** 2019-05-24

**Authors:** Soumitra Pathare

**Affiliations:** 1 Centre for Mental Health Law and Policy, Indian Law Society, Pune, Maharashtra, India

I have read the revised paper, the explanations for revisions provided by the authors and Prof Bartlett's comments on the revised version.

 The paper does raise important issues as highlighted by Prof Bartlett in his report on the revised version. For now though, I think the authors' have sufficiently addressed queries raised in my initial review and I am happy to approve it.

I have read this submission. I believe that I have an appropriate level of expertise to confirm that it is of an acceptable scientific standard.

# Peer review: Reviewer response for version 2

**DOI:** 10.21956/wellcomeopenres.16626.r35356

**Published:** 2019-04-30

**Authors:** Peter Bartlett

**Affiliations:** 1 School of Law and Institute of Mental Health, University of Nottingham, Nottingham, UK

I would provide some additional thoughts flowing from the paper and the comments provided by Dr Hindley, in hopes that they may assist in conceptualizing forthcoming elements of the project, most notably the qualitative arm. 

 My interest in particular is in the comments provided on what Dr Hindley labels as points 2 and 3 in my comments, notably as regards point 2:

 'In terms of the specific examples given, although an LPA may be the only *legal* way to designate a substitute decision maker, we should not assume that all service-users should want to operate on a legal level in this way. The same can be said of ADRTs. This is supported by the fact that 71% of people indicated a preference for informal Ads which suggests that a large proportion are actively seeking non-legal routes (although we accept the issues you raise with this definition and discuss these below).'

 and point 3:

 'The purpose of differentiating formal from informal in this way was to distinguish between people who had engaged or wanted to engage *knowingly *with the MCA and its associated legal framework from people who had engaged in ADM outside this legal framework. 

 'ADM is a complex area where the law interacts with medical practice, ethics and, ultimately, challenging and distressing moments in people’s lives. Although we agree that any form of ADM has the potential to be interpreted according to the MCA, we were aware that, for some people, this was not necessarily something they had considered when drafting their advance plan and, similarly, may not be something they would like to consider as part of their plan. The reasoning behind this was based on Morriss et al.’s survey which only addressed the MCA provisions for ADM. As our focus was on a broad definition of ADM, not limited to MCA.'

 These raise a forest of questions. 

 To begin with, what is meant in the comment by ‘legal’ in the response to point 2? If what is meant is that service users wanted a simple way to give people a real power to make decisions over them without signing an LPOA (perceived often and with some justification as a technical process, often involving a lawyer), fair enough – but that is not on offer at the moment. If the point is that the MCA should be amended to make this possible, that is certainly a debate that can be had.

 My use of the term ‘legal’ instead was meant to refer to whether the appointments were actually effective, and unless they are through an LPOA, at the moment they are not. ‘Informal’ schemes do not create substitute decision-makers; at best they create the purported decision-maker as ‘someone to be consulted on the matter in question’ [s 4(7)(a) – perhaps surprisingly, a provision we don’t see referred to much anywhere in the literature]. When the treatment decision gets made, it is still the clinician who makes it, and he or she must consider whether the treatment is in P’s best interests under s 4. If notwithstanding the view of the consultee the clinician thinks that it is in P’s best interest, the treatment should go forward, even if the consultee under 4(7)(a) disagrees, and the doctor exposes himself or herself to liability if it does not. In that situation, I suspect the person is likely to get treated, whatever the purported decision-maker identified by P thinks. If what the individual wants is to appoint somebody who has the actual authority to make the decisions in the event of incapacity so that in this example the substitute’s refusal holds sway, my original comment stands: the only way to do this at the moment is through an LPOA.

 I would encourage the team to explore some of these issues in the qualitative sessions: what exactly do people making ‘informal’ appointments of this type think they were doing? The status quo may, after all, be precisely the result that the individual wanted – formalized involvement of an identified person but not actually a transfer of decision-making. That is fine, and consistent with the existing Act, but it is not really appointment of a substitute decision-maker.

 That leads to the second part of the comment. I certainly take the point that a minority of people will have heard of the MCA in their daily lives, although it is much less clear to me why this is a helpful distinction for purposes of the present paper. To say that the MCA ‘may not be something they would like to consider as part of their plan’ is much more problematic. With very few exceptions (LPOAs and ADRTs that would result in the risk to life being the obvious examples), the MCA does not impose formalities requirements for statements: anything a person says may be relevant to a best interests determination under the MCA. It is a bit like saying ‘I do’ at the altar – the bride or groom may not want to think about the legal ramifications at that moment, but they are there nonetheless. I was a part of the team in the Morriss study cited, and I have to say that I disagree with the characterization that we ‘only addressed the MCA provisions for ADM’. In our study, almost two thirds of our 650 service user participants had not heard of the MCA before the study:  it was manifestly not something they had considered when drafting their advance plans (see Bartlett, Mudigonda, Chopra et al, 270 ^1^). What we did do is look at how those plans intersected with the provisions of the MCA. In the context of medical treatment, I am struggling to think of advance planning that would not be MCA relevant; perhaps the authors of the present paper can put me right on this.

 I suggest to the authors that the greater risk is people wishing to ignore or being unaware of the MCA relevance of their plans, when such relevance does exist. That too is a matter to which we drew attention (Bartlett, Mudigonda, Chopra et al, 274-5 ^1^). Again, it would be interesting to know from the qualitative elements of the present project how the people making plans view this set of issues.

 All of this moves the discussion a considerable way from the drafting of the present paper, and I am not sure that further re-drafting will be helpful in addressing the ongoing concerns noted above.  My initial concerns have been addressed I think as far as they can be given the data.  As for the above comments, suffice it to say that I look forward to reading the next stage of the research.

I have read this submission. I believe that I have an appropriate level of expertise to confirm that it is of an acceptable scientific standard.

## References

[1] BartlettPMudigondaMChopraAMorrissRJonesS: Planning for incapacity by people with bipolar disorder under the Mental Capacity Act 2005. *Journal of Social Welfare and Family Law*.2016;38(3) : 10.1080/09649069.2016.1228145 263-286 10.1080/09649069.2016.1228145

# Peer review: Reviewer response for version 1

**DOI:** 10.21956/wellcomeopenres.16350.r34711

**Published:** 2019-03-04

**Authors:** Soumitra Pathare

**Affiliations:** 1 Centre for Mental Health Law and Policy, Indian Law Society, Pune, Maharashtra, India

This paper reports the findings of a self-report online survey of attitudes of persons with Bipolar disorder towards Advance Decision making (ADM). 

 I liked the paper and it is an important addition to our understanding of this area. 

 I have two concerns which have been raised by two previous reveiwers and an additional concern of my own.

 First, issues of representativeness of the sample, both within the membership of BPUK and the broader population of persons with Biplolar Disorder who may not be members of BPUK. The former question (representativeness to membership of BPUK) can possibly be answered by the authors, as BPUK may have some demographic data on their members. The latter question is probably harder to address.

 The issue of representativeness is particularly important as the study had a high level of respondents with university education and a relatively high level of respondents who had some experience of ADM.

 The second issue of concern has also been flagged by one previous reviewer - the clarity of terms and questions in the questionnaire. For obvious reasons, this cannot now be changed, but it may be useful to take this into account when planning future studies using online self-report methodology.

 I am a little concerned by the authors mixing up concepts as are previously understood in the existing literature. Advance planning is an expression of a person's will and preference and does not (and should not) imply that a clinician or family member should be involved (although it maybe a person's choice to have them involved) and they should consent to this advance planning document.

 I am therefore concerned about this statement in the questionnaire:

 ““ *Would you like to collaborate with your psychiatrist or GP to produce an advance care plan that they could sign showing they agree with the contents of the plan?”*

 This should not be regarded as an advance planning document and is closer to a joint crisis plan. This question does not acknowledge the power dynamics in the patient-doctor relationship (especially in mental health due the presence of the Mental Health Act) and I am concerned that the authors would regard this as an advance planning document.

 Some minor quibbles:

 "Angst et al. reported a median of 0.4 and 0.3 episodes per year for BP I and II patients respectively" - For ease of reading can this be converted to years eg. 1 episode in 3 years etc.

 Can you include a glossary of all the acronyms - it does get confusing at times when reading the paper.

 And finally some self-promotion : you may want to read our review of bariers to advance decision making (see citation below).

I have read this submission. I believe that I have an appropriate level of expertise to confirm that it is of an acceptable scientific standard, however I have significant reservations, as outlined above.

## References

[1] ShieldsLSPathareSvan der HamAJBundersJ: A review of barriers to using psychiatric advance directives in clinical practice. *Adm Policy Ment Health*.2014;41(6) : 10.1007/s10488-013-0523-3 753-66 10.1007/s10488-013-0523-3 24248818

# Response

**Published:** 2019-04-04

**Authors:** Guy Hindley

**Affiliations:** King's College London SE1 1UL, UK

Thank you for your helpful comments. Please see our responses below:

  

1. *First, issues of representativeness of the sample, both within the membership of BPUK and the broader population of persons with Bipolar Disorder who may not be members of BPUK. The former question (representativeness to membership of BPUK) can possibly be answered by the authors, as BPUK may have some demographic data on their members. The latter question is probably harder to address.*
*The issue of representativeness is particularly important as the study had a high level of respondents with university education and a relatively high level of respondents who had some experience of ADM*.

 

 We agree this is an important limitation to our findings and have made several edits to emphasise this point, including demographic information on the population of people with BP in the UK. Please see our replies to Professor Craddock’s report above. 

*2.* *The second issue of concern has also been flagged by one previous reviewer - the clarity of terms and questions in the questionnaire. For obvious reasons, this cannot now be changed, but it may be useful to take this into account when planning future studies using online self-report methodology.*

 We agree that this study would have benefited with more clarity at the design stage. We will certainly learn from this for future studies. Please also see our reply to Peter Bartlett’s report above. 

*3.* *I am a little concerned by the authors mixing up concepts as are previously understood in the existing literature. Advance planning is an expression of a person's will and preference and does not (and should not) imply that a clinician or family member should be involved (although it maybe a person's choice to have them involved) and they should consent to this advance planning document.*

*I am therefore concerned about this statement in the questionnaire:*

**

*““Would you like to collaborate with your psychiatrist or GP to produce an advance care plan that they could sign showing they agree with the contents of the plan?”*

**

*This should not be regarded as an advance planning document and is closer to a joint crisis plan. This question does not acknowledge the power dynamics in the patient-doctor relationship (especially in mental health due the presence of the Mental Health Act) and I am concerned that the authors would regard this as an advance planning document*

 We agree that this statement is very much in line with the Joint Crisis Plan model. However, we disagree that this means it is therefore no longer an advance planning document, or alternately an advance decision. Although definitions may differ, our understanding of the term advance decision is a decision that is made in advance of someone losing the ability to make that decision for themselves. It therefore makes no judgement on how or with whom this decision is made so long as the individual is involved in that decision-making process. 

 If we utilise this definition, there can therefore be several different approaches to practicing ADM. Nicaise et al. provide an interesting analysis of the conceptual frameworks behind different psychiatric advance directives breaking it down into “user’s autonomy”, “therapeutic alliance” and “integration of care-partnership”. ^3^According to this approach, your definition would fall firmly under the “user’s autonomy” domain, while Joint Crisis Plans, and the sort of intervention hinted at in the question above would lean more to the “Therapeutic Alliance” domain. What’s more, we do make reference to this alternative model for ADMs in the text as you can see below:

*“Some have suggested that this is the way ADM should be conducted: for ADM to truly enhance autonomy, they should be completed without the involvement of mental health professionals” (P14)*

 This is not to say that the power dynamics within the doctor-patient relationship are not a key consideration to this kind of ADM, and perhaps an area that we have not explored sufficiently within our study. Nonetheless, we feel this is, at present, outside the scope of our paper and we remain of the opinion that the model proposed above is consistent with the broader definition for ADM that we and others have used.  

*4.* *"Angst et al. reported a median of 0.4 and 0.3 episodes per year for BP I and II patients respectively" - For ease of reading can this be converted to years eg. 1 episode in 3 years etc.*

 This has been changed accordingly. 

*5.* *Can you include a glossary of all the acronyms - it does get confusing at times when reading the paper.*

 We have removed several of the acronyms and also provided a glossary of the remaining acronyms. 

*6.* *And finally some self-promotion: you may want to read our review of bariers to advance decision making (see citation below).*

 Thank you for pointing us in the direction of your informative review. We hope our findings help elaborate further on many of the themes you have discussed. It was particularly helpful to read your discussion on revocability, enforceability and prior knowledge. We have expanded slightly our discussion on the latter to reflect this and referenced your review accordingly:

*“Low levels of awareness of the MCA has previously been reported among people with BP while limited awareness of ADM among people with mental illness including dementia has also been identified as a key barrier to its implementation* ^4^ *”*

 References:

 Nicaise P, Lorant V, Dubois V. Psychiatric Advance Directives as a complex and multistage intervention: a realist systematic review. *Health Soc Care Community*. 2013;21(1):1-14. doi:10.1111/j.1365-2524.2012.01062.

# Peer review: Reviewer response for version 1

**DOI:** 10.21956/wellcomeopenres.16350.r34712

**Published:** 2019-02-18

**Authors:** Peter Bartlett

**Affiliations:** 1 School of Law and Institute of Mental Health, University of Nottingham, Nottingham, UK

This article reports the results of an online survey of the attitudes of people with bipolar disorder (n=932) to various forms of advance decision-making in anticipation of a loss of mental capacity. The article is quantitative only: there is no qualitative analysis.

 There is much in this article to like. It would appear to be a robust and statistically astute survey, drawn from the contact list of Bipolar UK. Statistical techniques appear appropriate, and, in methodological terms, the findings seem convincing.

 I am happy enough with the sample size, but a bit more discussion might be appropriate as to its representativeness. I understand that this cannot be according to the classic statistical rules – it is not ‘random’ in that sense – but it would be helpful to know whether the demographics of the sample broadly match the demographics of the broader population of people with bipolar. I also note that fewer than 1/3 of the people who accessed the questionnaire finished it. As it stands, the response rate is only 5.6%, less almost 1/6 of responses that were excluded [p5]. Some indication of how typical this sample is of people with bipolar would be helpful.

 I think the article would be significantly improved if some of the concepts and categories were explained more carefully, and/or the apparent incoherences discussed more carefully.  On page 7, for example, we are told that 68% of respondents wanted to state who should make decisions for them in the event that they lost capacity, but only 40-43% would make an LPA, without any acknowledgement that the only legal way to do the former is to do the latter – that is what an LPA is, and without it, any view as to who should make decisions for you has no effect - it is still (for medical decisions) the treating physician. There is similarly nothing to suggest that the people who wished to refuse specific medications understood that the way you do that is through an ADRT (the numbers are similar for each - if they were the same people, that should be explained, but I would be surprised). I suspect what is going on here is that the respondents have limited and sometimes incorrect knowledge of the legal provisions. That is fair enough – normal people do not sleep with copies of the MCA under their pillows, and cannot readily parrot out its terms – but if in fact they do not really understand the provisions, and may misunderstand them in different ways, it raises questions as to how we interpret their responses. 

 The paper similarly speaks of ‘informal’ plans, as distinct (it would seem) from ADRTs and statements of wishes. It is not at all clear what this means. It is possible that some respondents read it as a distinction between oral and written statements, since the questionnaire refers to ADRTs and statements of wishes as ‘legal documents’. In this the questionnaire is deficient, since there is no requirement under the MCA that statements of wishes nor (with exceptions not relevant here) that ADRTs be in any particular form: in both cases quite informal statements, including oral statements, are meant to have legal effect. (I think there is a sensible argument to the effect that the MCA should not have taken that approach, but that is an issue for another time: that is the approach it takes.) At other times, though, the questionnaire seems to view advance care plans or crisis plans (presumably whether oral or in writing?) as ‘informal’, in juxtaposition to ADRTs and statements of wishes. Again, it is not at all clear what distinction is being drawn here, as wishes or treatment refusals contained in care plans or crisis plans are manifestly statements of wishes or ADRTs under the MCA. This lack of clarity again makes analysis of the results difficult: what did the people think they were agreeing to?

 Page 4 of the draft contains an explanation of what is meant by ‘self-binding directive’, a concept that is discussed at some length in the paper. There is a difficulty that the detail of this definition is not reflected in the only question about SBDs in the questionnaire, which refers only to ‘the person want[ing] the contents of their advance care plan to be respected even if they no longer agree with it when they are unwell.’  Again, I am not sure that one can slide between the two articulations in quite the way that the paper does.

 There is an ambiguity running throughout the questionnaire as to whether it is about treatment for mental health conditions or advance decision-making much more broadly. The questionnaire tends to phrase advance decision-making in the context of involvement by the care team and medical professionals, suggesting the former, while there are references in addition to much broader planning, albeit often placed in the context of a care plan (which is generally understood as a relatively medical mechanism). These distinctions are not always teased out as carefully as they might have been. Thus one might well imagine an affected person P wanting to discuss his or her medical treatment choices with medical and care professionals; it is much less obvious that he or she would, if running a business, want to discuss with medical and care professionals how the business should be run in the event of incapacity. The paper does seem to elide these differences at times, and I am afraid I did not find this convincing, and when respondents are discussing who they would seek advice from, it is not obvious which sort of decision they had in mind.

 These issues I think really are problematic, and it is difficult to see how they can be remedied since they flow from the questionnaire itself. The ambiguities do make it somewhat difficult to see how the analysis of the results should be understood, however.

 I have a number of lesser criticisms that the authors might wish to consider.

 In the discussion of the MCA and MHA, it would be worth noting that the MHA covers only treatment for mental disorder: If a person with bipolar has a somatic health disorder, the MCA applies.

 The discussion of ADs and their use in crisis situations is interesting but begs the question of the 50% of respondents who did not feel that their plan was respected in the crisis, the 40% that did not think they recovered faster, the 40% that did not report improved experience of health services, the 45% that were not happy with how their plan had been used in the crisis, the 56% that did not find that the plans were very or extremely helpful in reducing harm to relationships, the 66% that did not report a reduction in physical harm, and the 77% that did not find them very or extremely helpful in reducing overspending. It does feel rather that we are being told half a story here!

 The fact that after the statistical beeps and whistles get applied, the only correlation with support of SBDs is shown to be trust in medics is in a sense profoundly unsurprising – although still worth saying. It does return to the question of what is meant by an SBD, and medical contextualisation. Do we know whether they were thinking of medical SBDs here (as would be my guess) as distinct from SBDs, say, as to how a business will be run?

 Finally, I think that for a paper of this size, there is too much of a deluge of acronyms – BP, SMI, DMC-T, ADM, ADRT, EPA, UNCRPD (used only once in the body of the text), MHA, MCA, AS, SBD, BPUK. Are they all really necessary? Only about half will be known to people who work in the field; even for them the paper will therefore be hard going. For neophytes, this is likely to be a serious barrier.

I have read this submission. I believe that I have an appropriate level of expertise to confirm that it is of an acceptable scientific standard, however I have significant reservations, as outlined above.

# Response

**Published:** 2019-04-04

**Authors:** Guy Hindley

**Affiliations:** King's College London SE1 1UL, UK

Thank you for your detailed and insightful comments. Please see below for responses to the issues you have raised:

  

1. *I am happy enough with the sample size, but a bit more discussion might be appropriate as to its representativeness. I understand that this cannot be according to the classic statistical rules – it is not ‘random’ in that sense – but it would be helpful to know whether the demographics of the sample broadly match the demographics of the broader population of people with bipolar. I also note that fewer than 1/3 of the people who accessed the questionnaire finished it. As it stands, the response rate is only 5.6%, less almost 1/6 of responses that were excluded [p5]. Some indication of how typical this sample is of people with bipolar would be helpful*.

 

 This is certainly an important limitation that is discussed in depth in Professor Nick Crawford’s report above. As mentioned above, we are unfortunately unable to compare the demographics of our sample directly to the BPUK’s mailing list as they do not store this information. Nor are we able to compare the demographics of people who started the survey to people who finished the survey, which would also provide some insight into the response rate, as the demographics was at the end of the survey. Nonetheless your suggestion to compare our demographics to those of the wider population of people with bipolar is helpful. We have therefore added the following to our discussion:

*“This is a predominantly white (94%), female (71%), well-educated sample (64% university educated) which is not representative of the wider population of people with bipolar in the UK (88.8% white, 48.9% female  and 36% university educated)* *^1^* *.”*

*2. I think the article would be significantly improved if some of the concepts and categories were explained more carefully, and/or the apparent incoherences discussed more carefully.  On page 7, for example, we are told that 68% of respondents wanted to state who should make decisions for them in the event that they lost capacity, but only 40-43% would make an LPA, without any acknowledgement that the only legal way to do the former is to do the latter – that is what an LPA is, and without it, any view as to who should make decisions for you has no effect - it is still (for medical decisions) the treating physician. There is similarly nothing to suggest that the people who wished to refuse specific medications understood that the way you do that is through an ADRT (the numbers are similar for each - if they were the same people, that should be explained, but I would be surprised). I suspect what is going on here is that the respondents have limited and sometimes incorrect knowledge of the legal provisions. That is fair enough – normal people do not sleep with copies of the MCA under their pillows, and cannot readily parrot out its terms – but if in fact they do not really understand the provisions, and may misunderstand them in different ways, it raises questions as to how we interpret their responses. * 

 We attempted to prevent this by providing succinct definitions of each of the provisions for ADM currently available under the MCA at the beginning of the questionnaire (p2, supplementary material). This was presented on its own page on the online version which we hoped would encourage people to read these definitions. 

 Nonetheless, survey methodology is certainly vulnerable to validity problems due to misunderstanding of questions. We had previously made reference to this in the limitations section, but we have made this more explicit as follows: 

*“ What’s more it is possible that some participants misunderstood some of the complex concepts contained within the questionnaire.”*

 However, it is important to note that misunderstanding is not the only possible explanation for these inconsistencies. In terms of the specific examples given, although an LPA may be the only *legal*way to designate a substitute decision maker, we should not assume that all service-users should want to operate on a legal level in this way. The same can be said of ADRTs. This is supported by the fact that 71% of people indicated a preference for informal ADs which suggests that a large proportion are actively seeking non-legal routes (although we accept the issues you raise with this definition and discuss these below). This is further supported by unpublished findings from focus groups that we have conducted which suggest that ambiguous attitudes towards legally-binding ADM is a real finding, not just an artefact of our survey design.

 We discuss this ambiguity in the section “ *Inconsistent attitudes towards legal status”.  *We have expanded this section as follows to include the two further examples that you have suggested:

*“Respondents expressed a preference for non-legally binding formsof ADM which included information about refusing medication or substitute decision makersover legally binding forms that could communicate the sameinformation such asADRTs and LPAs.”*

 Unfortunately the number of people wishing to refuse specific medication (68%) was significantly higher than those who wanted to make an ADRT (35%) and so the additional analysis suggested was not informative.

*3. The paper similarly speaks of ‘informal’ plans, as distinct (it would seem) from ADRTs and statements of wishes. It is not at all clear what this means. It is possible that some respondents read it as a distinction between oral and written statements, since the questionnaire refers to ADRTs and statements of wishes as ‘legal documents’. In this the questionnaire is deficient, since there is no requirement under the MCA that statements of wishes nor (with exceptions not relevant here) that ADRTs be in any particular form: in both cases quite informal statements, including oral statements, are meant to have legal effect. (I think there is a sensible argument to the effect that the MCA should not have taken that approach, but that is an issue for another time: that is the approach it takes.) At other times, though, the questionnaire seems to view advance care plans or crisis plans (presumably whether oral or in writing?) as ‘informal’, in juxtaposition to ADRTs and statements of wishes. Again, it is not at all clear what distinction is being drawn here, as wishes or treatment refusals contained in care plans or crisis plans are manifestly statements of wishes or ADRTs under the MCA. This lack of clarity again makes analysis of the results difficult: what did the people think they were agreeing to?* 

 The purpose of differentiating formal from informal in this way was to distinguish between people who had engaged or wanted to engage *knowingly*with the MCA and its associated legal framework from people who had engaged in ADM outside this legal framework. 

 ADM is a complex area where the law interacts with medical practice, ethics and, ultimately, challenging and distressing moments in people’s lives. Although we agree that any form of ADM has the potential to be interpreted according to the MCA, we were aware that, for some people, this was not necessarily something they had considered when drafting their advance plan and, similarly, may not be something they would like to consider as part of their plan. The reasoning behind this was based on Morriss et al.’s survey which only addressed the MCA provisions for ADM. As our focus was on a broad definition of ADM, not limited to MCA.

 Nonetheless, we agree that this distinction was not made clear in the write-up and we can understand why this may have been confusing. We have therefore added the following to the introduction:

 “ *If ADM is to be expanded amongst the BP community, it is important to refine our understanding of the views of those with BP and how existing provision might be tailored to meet the needs of those using it. In particular, it is possible that people with BP may be practicing ADM without awareness of the MCA. In order to make this distinction, we have referred to “informal ADM” to mean advance plans that were created outside of the MCA framework for advance planning*.”

 In the process of doing this we accept that our definitions at the beginning of the questionnaire are simplified and do not capture the nuances of the MCA. In particular, we agree that ADRTs and ASWFs could have been communicated verbally. This is clearly one of the important limitations to survey methodology when tackling such a complicated and nuanced topic: simplifications are hard to avoid. We hope that our focus-group work and analysis of the free text boxes will help to triangulate these findings. We have discussed this in the limitations as follows:

*“What’s more it is possible that some participants misunderstood some of the complex concepts contained within the questionnaire….*Further structured qualitative analysis of the free text boxes included in the survey will be reported in a future publication to help deepen our understanding of the respondents’ views.” 

 4. *Page 4 of the draft contains an explanation of what is meant by ‘self-binding directive’, a concept that is discussed at some length in the paper. There is a difficulty that the detail of this definition is not reflected in the only question about SBDs in the questionnaire, which refers only to ‘the person want[ing] the contents of their advance care plan to be respected even if they no longer agree with it when they are unwell.’  Again, I am not sure that one can slide between the two articulations in quite the way that the paper does.* 

 We agree that the questionnaire item relating to SBDs is a simplified version of the discussion of SBDs as given in the introduction. Nonetheless we feel this does tackle the central concept at the heart of self binding: *the concept that the individual wants the contents of their plan to be respected even if they no longer agree to it when they are unwell*. We were wary of providing a lengthier ethical or legal definition given the nature of surveys. This was discussed during the piloting phase and our pilots agreed with this decision. 

 Nonetheless, we accept that it may be misleading to use the same term for both the discussion of SBDs given in the introduction and the item which we use in the survey. As this is an issue of how the information from the questionnaire is represented in the paper rather than incoherencies within the questionnaire itself, we feel this can be remedied. In order to make the distinction we have added the following to the introduction:

*“However, at the heart of both accounts is a willingness to engage with the idea of “self-binding” – the concept that the individual wants the contents of their plan to be respected even if they no longer agree to it when they are unwell.”*

 We have subsequently referred to “self-binding” rather than SBDs in the results and discussion whenever referring to this specific item. 

 Despite this, the outcome of interest relating to SBDs combines this item with an item about willingness to collaborate with a doctor. We think this is justified as these are, according to Gergel and Owen’s model, the two essential components of SBD. We have made this more explicit in the methods by adding the following:

*“ This was designed as a proxy measure for interest in self binding directives as these are essential components to the model as proposed by Gergel and Owen* ^2^ *.”*

 We have also added the following paragraph in the limitations:

*“ We utilised a proxy-measure for interest in SBDs by combining interest in self-binding statements with a willingness to collaborate with doctors. This was because we decided that attempting to explain SBDs in a survey was likely to generate confusion. This means our results cannot be interpreted as a direct endorsement for SBDs. We have conducted focus groups in which SBDs were discussed as part of a parallel study which will help to triangulate these findings.”*

  5. *There is an ambiguity running throughout the questionnaire as to whether it is about treatment for mental health conditions or advance decision-making much more broadly. The questionnaire tends to phrase advance decision-making in the context of involvement by the care team and medical professionals, suggesting the former, while there are references in addition to much broader planning, albeit often placed in the context of a care plan (which is generally understood as a relatively medical mechanism). These distinctions are not always teased out as carefully as they might have been. Thus one might well imagine an affected person P wanting to discuss his or her medical treatment choices with medical and care professionals; it is much less obvious that he or she would, if running a business, want to discuss with medical and care professionals how the business should be run in the event of incapacity. The paper does seem to elide these differences at times, and I am afraid I did not find this convincing, and when respondents are discussing who they would seek advice from, it is not obvious which sort of decision they had in mind.* 

 As you rightly point out, our questionnaire has an implicit focus on mental health treatment and there are whole domains of advance planning relating to financial affairs, work and relationships that we have not fully addressed. For our reader’s sake, we have made this more explicit by including the following to the introduction:

*“ It can also contain information about various aspects of an individual’s life such as medical treatment, financial affairs or arrangements for work. This study focuses primarily on medical treatment.”*

 And adding the following to the Aims:

*“This study aimed to address the following objectives, with a focus on medical treatment:”*

 This does not, however, address the ambiguity within the questionnaire itself. We have therefore made reference to this in the limitations section, as well as the fact that we do not explore other forms of ADM as follows:

*“ What’s more it is possible that some participants misunderstood some ofthe complex concepts contained within the questionnaire. Our focus on ADM in relation to medical treatment may have added to this potential misunderstanding. ADM can relate to financial or work matters and it is possible that some of our respondents may have been referring to these in some of their responses. Further structured qualitative analysis of the free text boxes included in the survey will be reported in a future publication, helping to disambiguate this.While further research is needed to explore ADM for matters such as finances and work.”*

  6. *These issues I think really are problematic, and it is difficult to see how they can be remedied since they flow from the questionnaire itself. The ambiguities do make it somewhat difficult to see how the analysis of the results should be understood, however*. 

 We hope we have addressed these concerns by drawing attention to the broader issue of misunderstanding between participants and researchers in the limitations section, clarifying our definitions of “informal ADM” and the self-binding item and making our medical focus more explicit. We are therefore confident that our findings remain sufficiently valid for us to draw important, if tentative, conclusions, despite these limitations. 

 7. *In the discussion of the MCA and MHA, it would be worth noting that the MHA covers only treatment for mental disorder: If a person with bipolar has a somatic health disorder, the MCA applies.* 

 Thanks for picking up on this. We do state that the MHA *“enables compulsory treatment for mental disorder” (p3). *However to make this more explicit we have added the following:

*“Although if they were to be treated for a physical health condition, the MCA would still apply.”*

*8. The discussion of ADs and their use in crisis situations is interesting but begs the question of the 50% of respondents who did not feel that their plan was respected in the crisis, the 40% that did not think they recovered faster, the 40% that did not report improved experience of health services, the 45% that were not happy with how their plan had been used in the crisis, the 56% that did not find that the plans were very or extremely helpful in reducing harm to relationships, the 66% that did not report a reduction in physical harm, and the 77% that did not find them very or extremely helpful in reducing overspending. It does feel rather that we are being told half a story here!* 

 This was in fact a discussion we had during the drafting of the paper and we decided it was a good example of the “glass half full vs glass half empty” debate! The point we are trying to make here is that even the fact that, for example, 50% of ADMs were highly or completely respected, this is a notable finding given the scepticism around the implementation of ADM in clinical practice. This is therefore worth stressing. Nonetheless, we agree that this could be misleading and so we have added the following:

*“This is preliminary evidence that ADM produced outside of an “experimental” setting canbe used in crisis with therapeutic effect and that they tend to be respected when they are used, although it is worth noting that significant proportions of our sample did not report these positive experiences.”*

 In addition, there is in fact a lot less than half of the story being told here as there is only so much we can understand by these quantitative survey responses. We hope to reveal much more of the detail underlying these responses during the analysis of the free text boxes.  

  9. *The fact that after the statistical beeps and whistles get applied, the only correlation with support of SBDs is shown to be trust in medics is in a sense profoundly unsurprising – although still worth saying. It does return to the question of what is meant by an SBD, and medical contextualisation. Do we know whether they were thinking of medical SBDs here (as would be my guess) as distinct from SBDs, say, as to how a business will be run?* 

 Although this finding may seem obvious, the debate over the role of medical practitioners in the production of ADM documents is ongoing (as can be seen in Prof Pathare’s report below). This finding may, therefore, be more surprising to people who would advocate a patient driven SBD motivated, for example, by distrust that doctors deliver continuity of care based on prior knowledge of them.

 The fact that trust in medics *was*significantly associated with interest in SBDs would suggest that a sufficient number of respondents understood that we were talking about medical SBDs and we think the effect of any ambiguity in the questionnaire would be to underestimate the strength of this association. 

*10. Finally, I think that for a paper of this size, there is too much of a deluge of acronyms – BP, SMI, DMC-T, ADM, ADRT, EPA, UNCRPD (used only once in the body of the text), MHA, MCA, AS, SBD, BPUK. Are they all really necessary? Only about half will be known to people who work in the field; even for them the paper will therefore be hard going. For neophytes, this is likely to be a serious barrier.* 

 On re-reading the paper after some time away, I couldn’t agree more with you on this point! As Professor Pathare suggested, we have therefore added a glossary of terms to make this a little less daunting. We have also removed the following acronyms: SMI, DMC-T, DMC, UNCRPD and AS.

# Peer review: Reviewer response for version 1

**DOI:** 10.21956/wellcomeopenres.16350.r34714

**Published:** 2019-02-12

**Authors:** Nick Craddock

**Affiliations:** 1 Institute of Psychological Medicine & Clinical Neurosciences, Cardiff University, Cardiff, UK

This paper describes a self-report questionnaire study about experiences of, and attitudes towards, advance decision making (ADM) in individuals with bipolar disorder. The sampling frame population is the Bipolar UK charity mailing list. The response rate is quoted as 5.61% of those on that list but the main analyses presented relate to self-reported patients and excludes self-reported carers - a total of 932 respondents out of 20,134 people who had registered their email addresses to be contacted by the charity.

 The authors report various summary statistics that demonstrate that many in their sample has an interest in ADM and that their access to experience of ADM falls short of their ideal wish. The authors state that this is the largest study to date of this issue and is a "leap forward" in understanding.

 The use of ADM in bipolar disorder is an issue that has complicated ethical and practical issues that require attention and it is helpful to have research that helps to inform these discussions.  However, there are substantial limitations with the current study that greatly reduce the ability to make any generalisations or broad conclusions.

 The fundamental limitation is the difficulty in knowing to what extent the sample studied relates to the population of individuals with a bipolar disorder in the UK. Their respondents are a very small proportion of those on the email contact list of the charity bipolar UK who have self identified as having a bipolar diagnosis (who are in turn a subset of the broader membership of bipolar UK) - it would be helpful to know how the demographics of those on the email list as a whole compare with those in sample studied.

 Those who chose to volunteer for the questionnaire study are likely to be substantially biased towards those who have particular views they would like to articulate - and, of course, the authors acknowledge the high level of university-level education. 

 Of course, the study shows that there are individuals who want to use ADM and that people have experiences of ADM that are inadequate or unavailable. However, it is not straightforward, with the data and and analyses presented to know how this may generalise to the wider population of those with bipolar disorder diagnoses within the UK.

 The claim that this is a "leap forward" seems inappropriately hyperbolic. This is a useful first step to looking at the issue. However, wording such as "suggests the need for more systematic study" would be much more academically circumspect, together with a more detailed discussion about the difficulties in generalising. It would be helpful if the authors could indicate what they would consider a study design that would provide more systematic and generalisable data that would give robust conclusions.

 In summary - an interesting preliminary study of an important topic but substantial caution is required in generalising from findings because of the low response rate and the sample biases.

I have read this submission. I believe that I have an appropriate level of expertise to confirm that it is of an acceptable scientific standard, however I have significant reservations, as outlined above.

# Response

**Published:** 2019-04-04

**Authors:** Guy Hindley

**Affiliations:** King's College London SE1 1UL, UK

Dear Professor Craddock,

 Thank you for your thoughtful comments. Please see below for responses to the issues raised in your report:

*1.* *The fundamental limitation is the difficulty in knowing to what extent the sample studied relates to the population of individuals with a bipolar disorder in the UK. Their respondents are a very small proportion of those on the email contact list of the charity bipolar UK who have self identified as having a bipolar diagnosis (who are in turn a subset of the broader membership of bipolar UK) - it would be helpful to know how the demographics of those on the email list as a whole compare with those in sample studied.*

 We agree that this is an important limitation to our study. In the limitations section we do note this ourselves: 

*“The use of BPUK mailing list as the sampling frame and a low response rate introduce further selection bias. People with a prior awareness or interest in ADM are likely to be over-represented. These findings must therefore be generalised with caution and the study seen as exploratory.”*

 However we accept that it would be helpful to also include it in the concluding paragraph. We have therefore added the following sentence to the conclusions:

*“The results suggest services users with BP want to use ADM and they want collaborative input from mental health services in doing so. However, the low response rate mean further  research is needed to determine the generalisability of these findings.”*

 We also agree that comparing responders to non-responders would allow us to assess how representative our sample is of the demographics of the BPUK mailing list (although this would still not address the issue of selection bias towards people with a prior interest in ADM). Unfortunately, BPUK do not keep this kind of demographic information about the members of their mailing list and so this was not possible. 

*2.* *Of course, the study shows that there are individuals who want to use ADM and that people have experiences of ADM that are inadequate or unavailable. However, it is not straightforward, with the data and and analyses presented to know how this may generalise to the wider population of those with bipolar disorder diagnoses within the UK.*

 We accept that given the methodology used and the response rate achieved, it is very difficult to comment on the generalisability of these findings. In the limitations section we currently say:

*“These findings must therefore be generalised with caution and the study seen as exploratory.”*

 To make this limitation more explicit we have edited this sentence as follows:

*“It is therefore difficult to generalise these findings and the study should be seen as exploratory.”*

 Nonetheless, as we note in the limitations section, the results of this survey do still represent the opinions and experiences of almost 1000 people with bipolar in the UK. Although these findings may not be generalisable to the entire population, they are likely to represent a subset of this population. Given the dearth of research on this topic within this population, particularly with regards to *experiences *of ADM, we feel our findings do still represent an important step forward in the field’s understanding.

*3.* *The claim that this is a "leap forward" seems inappropriately hyperbolic. This is a useful first step to looking at the issue. However, wording such as "suggests the need for more systematic study" would be much more academically circumspect, together with a more detailed discussion about the difficulties in generalising. It would be helpful if the authors could indicate what they would consider a study design that would provide more systematic and generalisable data that would give robust conclusions.*

 We agree that, given the limitations, it would be preferable to change the language used here. We have therefore changed this sentence to read:

*“It provides an important step forward in understanding experiences and attitudes towards ADM in this group.”*

 With regard to the discussion of generalisation and study design we have already mentioned respondent-driven sampling which can be utilised in hard to reach groups and given a reference (reference 36). In addition to the changes already discussed above, we have also indicated a study design that could help provide more generalisable findings in clinical settings, as suggested:

*“ While in order to understand the generalisability of these findings, a simplified version of the survey should be conducted using a more representative sampling technique such as convenience sampling through outpatient clinics or systematic sampling through the use of a patient research database such as the Clinical Record Interactive Search system”*
